# Additional Saturday rehabilitation improves functional independence and quality of life and reduces length of stay: a randomized controlled trial

**DOI:** 10.1186/1741-7015-11-198

**Published:** 2013-09-10

**Authors:** Casey L Peiris, Nora Shields, Natasha K Brusco, Jennifer J Watts, Nicholas F Taylor

**Affiliations:** 1 Department of Physiotherapy, La Trobe University, Melbourne, Victoria, Australia; 2 Allied Health Clinical Research Office, Eastern Health, Box Hill, Victoria 3128, Australia; 3 Allied Health Learning and Research Unit, Northern Health, Bundoora, Victoria, Australia; 4 Physiotherapy Services, Cabrini Health, Malvern, Victoria, Australia; 5 School of Health and Social Development, Faculty of Health, Deakin University, Burwood, Victoria, Australia

**Keywords:** Occupational therapy, Physiotherapy, Rehabilitation, Quality of life

## Abstract

**Background:**

Many inpatients receive little or no rehabilitation on weekends. Our aim was to determine what effect providing additional Saturday rehabilitation during inpatient rehabilitation had on functional independence, quality of life and length of stay compared to 5 days per week of rehabilitation.

**Methods:**

This was a multicenter, single-blind (assessors) randomized controlled trial with concealed allocation and 12-month follow-up conducted in two publically funded metropolitan inpatient rehabilitation facilities in Melbourne, Australia. Patients were eligible if they were adults (aged ≥18 years) admitted for rehabilitation for any orthopedic, neurological or other disabling conditions excluding those admitted for slow stream rehabilitation/geriatric evaluation and management. Participants were randomly allocated to usual care Monday to Friday rehabilitation (control) or to Monday to Saturday rehabilitation (intervention). The additional Saturday rehabilitation comprised physiotherapy and occupational therapy. The primary outcomes were functional independence (functional independence measure (FIM); measured on an 18 to 126 point scale), health-related quality of life (EQ-5D utility index; measured on a 0 to 1 scale, and EQ-5D visual analog scale; measured on a 0 to 100 scale), and patient length of stay. Outcome measures were assessed on admission, discharge (primary endpoint), and at 6 and 12 months post discharge.

**Results:**

We randomly assigned 996 adults (mean (SD) age 74 (13) years) to Monday to Saturday rehabilitation (n = 496) or usual care Monday to Friday rehabilitation (n = 500). Relative to admission scores, intervention group participants had higher functional independence (mean difference (MD) 2.3, 95% confidence interval (CI) 0.5 to 4.1, *P* = 0.01) and health-related quality of life (MD 0.04, 95% CI 0.01 to 0.07, *P* = 0.009) on discharge and may have had a shorter length of stay by 2 days (95% CI 0 to 4, *P* = 0.1) when compared to control group participants. Intervention group participants were 17% more likely to have achieved a clinically significant change in functional independence of 22 FIM points or more (risk ratio (RR) 1.17, 95% CI 1.03 to 1.34) and 18% more likely to have achieved a clinically significant change in health-related quality of life (RR 1.18, 95% CI 1.04 to 1.34) on discharge compared to the control group. There was some maintenance of effect for functional independence and health-related quality of life at 6-month follow-up but not at 12-month follow-up. There was no difference in the number of adverse events between the groups (incidence rate ratio = 0.81, 95% CI 0.61 to 1.08).

**Conclusions:**

Providing an additional day of rehabilitation improved functional independence and health-related quality of life at discharge and may have reduced length of stay for patients receiving inpatient rehabilitation.

**Trial registration:**

Australian and New Zealand Clinical Trials Registry ACTRN12609000973213

Please see related commentary: http://www.biomedcentral.com/10.1186/1741-7015-11-199.

## Background

Rehabilitation involves specialized, coordinated, multidisciplinary care that aims to restore functional independence in physical and cognitive activities [1]. Allied health services are commonly provided as part of a multidisciplinary team during inpatient rehabilitation with physiotherapy and occupational therapy services being the most frequently required [2]. There is evidence that multidisciplinary rehabilitation is effective [3-6], so the question is not ‘what’ should be provided during rehabilitation but ‘how much’ should be provided to lead to the most efficient gains in functional independence during rehabilitation [7].

There has been recent debate in the UK about providing 7-day acute healthcare in the National Health Service [8,9]. It has been noted that in such a complex healthcare system, one area cannot work effectively at the weekend without having access to other areas that must also be functioning at the weekend [8,9]. Recent debate has centered on consultant and elective medical care, but rehabilitation services also need to be considered as they are an important part of the healthcare system.

Despite the view that most hospitals provide weekend rehabilitation, only 30% of rehabilitation hospitals in Australia offer weekend therapy [10]. Although weekend allied health services are more common in acute hospitals in the UK, Western Europe, Canada and Australia [10-12], staffing is reduced by up to 88% on weekends compared to weekdays, and is offered only to patients at risk of deterioration or those being discharged over the weekend [11]. A possible explanation for the limited amount of weekend therapy being provided is the lack of evidence to support it. A recent retrospective study found that 7 days per week of rehabilitation did not improve function, but reduced length of stay by 1 day compared to 5 days per week of rehabilitation [13]. Another study indicated that additional Saturday physiotherapy may reduce length of stay during rehabilitation [14] but was underpowered and did not include any other members of the multidisciplinary team or follow-up. Health service providers require quality evidence to determine whether weekend therapy is beneficial for all rehabilitation patients before they can decide whether to staff a full weekend service.

The primary aim of this study was to determine what effect providing an additional Saturday rehabilitation service in inpatient rehabilitation had on the discharge outcomes of functional independence, quality of life and length of stay. The secondary aim was to investigate if any benefits of providing additional therapy were maintained at 6 and 12 months after discharge from inpatient rehabilitation.

## Methods

### Design

This was a multicenter, single-blind, randomized controlled trial. The trial was registered with the Australian and New Zealand Clinical Trials Registry (ACTRN12609000973213) prior to patient recruitment. The trial was conducted according to the published trial protocol [15]. The only significant variations to the protocol related to the management of missing data, as described below in the data analysis section, and that the number of participants recruited exceeded the estimated sample. Ethics approval was received from University and Health Service Human Ethics Committees and written informed consent was provided by all participants.

### Settings

The trial took place at 2 publically funded metropolitan rehabilitation facilities (Angliss Hospital and Peter James Centre) with a combined total of 90 rehabilitation beds (providing multidisciplinary inpatient rehabilitation services in eastern metropolitan Melbourne, Australia). Recruitment occurred from 1 July 2010 to 30 June 2011. In Australia, patients admitted for rehabilitation are usually not able to return directly home from acute hospital due to reduced functional independence. Before being accepted for inpatient rehabilitation, patients are typically assessed in an acute hospital as being able to participate actively in rehabilitation with the expectation that they will improve sufficiently to return to community living.

### Participants

Participants were included if they were aged 18 years or older and had been admitted for rehabilitation at either of the two facilities. Participants with any orthopedic (e.g. fractures, elective joint replacements), neurological (e.g. stroke, multiple sclerosis, Parkinson disease) or other disabling condition (cardiac, pulmonary, deconditioning) were included. Participants were excluded if they were admitted for slow-stream rehabilitation termed ‘geriatric evaluation and management’ (as this patient group are managed differently to patients admitted for standard rehabilitation) or if they were enrolled in another intervention trial. Participants were not excluded if their primary language was a language other than English (an accredited interpreter assisted with informed consent and outcome measurement) or if they had reduced cognition (the next of kin was approached for informed consent).

### Randomization procedure

Participants were randomized to the intervention or the control group using a concealed method, with 1:1 allocation. The block allocation sequence was generated electronically and assignments concealed in sequentially numbered, sealed, opaque envelopes. Only after the participant was enrolled in the trial and had completed baseline testing was assignment made by opening the next envelope in the sequence. A member of the research team who was not involved in recruitment, assessment or treatment of participants prepared the envelopes.

### Intervention

Usual care rehabilitation was provided to all participants in both groups daily from Monday to Friday. Rehabilitation therapy focused on task-specific training and discharge planning and was at the discretion of the treating therapist. Patients at the two facilities usually receive about 2 h of physiotherapy and occupational therapy per weekday as well as full nursing, medical and other allied health services.

In addition, the intervention group was scheduled to receive a full physiotherapy and occupational therapy service on Saturday (an additional 1 h of each therapy). Weekend therapists may or may not have been the patient’s usual therapist but were therapists employed by the hospital network and not research staff. The content of the therapy provided at the weekend was similar to that which was provided during the week as determined by the patient’s Monday to Friday therapists. Instructions were provided by a written handover.

### Outcome measures

Outcome measures were assessed directly at admission and discharge and by telephone at 6 months and 12 months. The primary endpoint was assessment at discharge with follow-up measures of functional independence and health-related quality of life at 6 months and 12 months. Outcome assessors who measured primary and secondary outcomes were blinded to group allocation. The success of blinding was evaluated at the discharge assessment by asking assessors to guess their patient’s group allocation. Treating therapists and other members of the rehabilitation team (who made decisions regarding discharge) were not blinded to group allocation.

### Primary outcomes

Functional independence was assessed using the functional independence measure (FIM) [16] administered by credentialed assessors. The FIM consists of 18 items in 2 domains: motor (13 items) and cognitive (5 items). Each item is rated on a 7-point scale, where 1 reflects complete dependence and 7 reflects complete independence. Scores range from 18 (lowest function) to 126 (highest function). The FIM self-care score refers to items 1 to 6, which relate to feeding, grooming and dressing. The FIM mobility score refers to items 9 to 13, which relate to transfers, walking and stairs. The FIM has demonstrated strong psychometric properties in rehabilitation settings with good reliability (intraclass correlation coefficient (ICC) = 0.99) [17] and evidence of responsiveness and validity as a global disability measure for patients receiving rehabilitation [18]. An increase in FIM of 22 points or more is considered to reflect a clinically significant improvement in functional independence [19].

Health-related quality of life was assessed using the EuroQoL questionnaire (EQ-5D) and visual analog scale (EQ-VAS) [20]. The EQ-5D rates five domains of health including mobility, self-care, usual activities, anxiety/depression and overall health status, scores for which can be converted into a utility index score by using data from the general population [21]. The EQ-5D utility index has been used in a range of health conditions and changes in EQ-5D are correlated with changes in condition-specific measures [22]. A change in the EQ-5D utility index score of half a standard deviation was considered clinically significant [23].

Length of stay was measured as the number of overnight stays in the rehabilitation facility and was included as a primary outcome based on pilot data [14] that suggested patients who received additional Saturday therapy were discharged earlier but at a similar functional level to patients who received Monday to Friday therapy.

### Secondary outcomes

Secondary outcome measures included the Personal Care Participation Assessment and Resource Tool (PC-PART) [24], 10-m walk test [25,26], and the timed up and go test [27]. The modified Motor Assessment Scale [28] was completed by patients with stroke. Discharge destination was categorized as ‘same’ if participants returned to their usual place of residence or ‘worse’ if participants were unable to return home because they required more supported accommodation on discharge. The need for follow-up physiotherapy or occupational therapy on discharge was recorded.

Adverse events, including falls, skin tears and infections were recorded using the health services incident reporting database. Adverse events were classified as severe, moderate, mild, or no harm.

### Other outcome measures

Health service utilization and costs for participants in this trial will be reported elsewhere. Subsets of participants enrolled in the current trial had additional measures taken to explore the effects of additional rehabilitation. Physical activity levels were monitored [29,30] and in-depth interviews were conducted [31] on subsets of participants.

### Sample size

Based on one of the primary outcome measures (length of stay), a sample size of 712 participants was estimated in the trial protocol [15]. To recruit this number of participants a recruitment period of 18 months was anticipated.

### Statistical analysis

Analysis of covariance (ANCOVA) was used to analyze between-group differences in discharge (primary endpoint), 6-month and 12-month scores with baseline scores as covariate [32]. Intention to treat analysis, based on original group allocation, was used with any missing primary outcome data imputed using multiple imputation methods [33]. We assumed data were missing at random and used linear imputation for the continuous variables of length of stay, FIM, EQ-5D and EQ-VAS at admission, discharge, 6 months and 12 months via chained equations imputation generating five imputed datasets. In the trial protocol we specified that we would use the last value carried forward method [34]. Since the trial protocol was written, it has been recommended that multiple imputations may be a more appropriate method of dealing with missing data as it is less subject to bias [33,35]. The multiple imputation method was therefore chosen for dealing with missing data in this trial. For secondary outcomes, available data of all participants who were allocated were included in analyses without any imputation for missing data. Absolute risks, relative risks and number needed to treat (NNT) were calculated for the number of participants in each group who achieved clinically significant improvements, using the threshold values specified above, in primary outcome measures, returned to their usual accommodation, and required follow-up allied health therapy. A negative binomial regression model was used to analyze adverse events [36].

## Results

**Figure 1:**
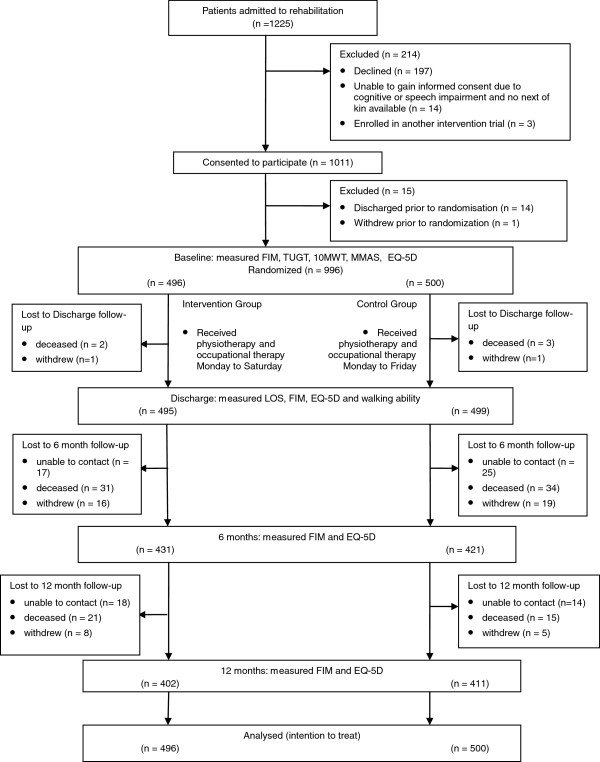
Flow of participants through the trial.

Over a 12-month period 1,225 eligible patients were admitted to rehabilitation at the 2 sites. A total of 996 patients provided informed consent to participate and were randomized to receive either Monday to Saturday rehabilitation (intervention) (n = 496) or Monday to Friday rehabilitation (control) (n = 500). Recruitment rates were higher than originally expected and the project steering committee decided to stop recruitment earlier than planned as it appeared that the target sample size would be reached prior to 18 months. Without any interim analyses being performed, it was decided to stop recruitment at 12 months. The primary outcome measure of length of stay was obtained for all participants (100%) on discharge. By the end of the trial (12-month follow-up) 106 participants had died (intervention group n = 54, control group n = 52). In all, 86% of participants (852 of 996) were available for follow-up at 6 months and 82% (813 of 996) at 12 months (Figure 1). Overall, 94% of primary outcome data was complete at discharge, 82% at 6 months and 79% at 12 months.

### Participants

Participants had a mean (SD) age of 74 (13) years and 637 (64%) were women (Table 1). A total of 579 (58%) participants were admitted with an orthopedic diagnosis, 203 (20%) with a neurological diagnosis and 214 (21%) participants were admitted with other disabling impairments. A total of 94% of participants were living independently in the community prior to their acute hospital admission.

**Table 1:** Baseline characteristics

**Characteristic**	**Randomized (n = 996)**	
	**Intervention (n = 496)**	**Control (n = 500)**
Age in years, mean (SD)	75 (13)	74 (13)
Age group, n (%)		
≤59 years	63 (13)	72 (14)
60 to 79 years	236 (48)	234 (47)
≥80 years	197 (40)	194 (39)
Gender, n male (%)	188 (38)	171 (34)
Diagnosis category, n (%)		
Stroke	81 (16)	79 (16)
Other neurological conditions	20 (4)	23 (5)
Orthopedic conditions	283 (57)	296 (59)
Pain syndromes	24 (5)	19 (4)
Cardiac/Pulmonary	25 (5)	23 (5)
Other disabling impairments	63 (13)	59 (12)
Functional independence (FIM)		
Total, mean (SD)	83 (20)	83 (21)
Mobility component, mean (SD)	16 (7)	16 (7)
Self-care component, mean (SD)	27 (8)	27 (8)
Cognitive component, mean (SD)	31 (6)	31 (6)
Health-related quality of life		
EQ-5D utility index, mean (SD)	0.32 (0.35)	0.37 (0.35)
Visual analog scale (0 to 100 mm), mean (SD)	57 (21)	56 (22)
Charlson comorbidity index, mean (SD)	1 (1)	1 (1)
Living independently in the community prior to admission, n (%)	466 (94)	466 (93)

Intervention = Monday to Saturday rehabilitation, control = Monday to Friday rehabilitation.

*EQ-5D* EuroQoL five dimensions questionnaire.

### Intervention

Participants in the intervention group received a mean of 53 more minutes of rehabilitation therapy (95% CI 31 to 74) per week compared to the control group. A total of 457 (92%) participants in the intervention group and 8 (2%) participants in the control group received at least 1 session of additional Saturday rehabilitation.

From available data, assessors correctly guessed group allocation on discharge 55% of the time.

### Effects of intervention

#### Functional independence

Participants in the intervention group had higher FIM scores on discharge (mean difference (MD) 2.3, 95% CI 0.5 to 4.1, *P* = 0.01), and possibly at 6 months (MD 2.0, 95% CI 0.0 to 4.0, *P* = 0.05), but not at 12 months (MD 1.3, 95% CI −0.9 to 3.5, *P* = 0.24) compared to the control group (Table 2). Participants in the intervention group were 17% more likely to achieve a clinically significant improvement in FIM of at least 22 FIM points at discharge (Risk Ratio (RR) 1.17, 95% CI 1.03 to 1.34) compared to those in the control group. For every 13 patients provided with the intervention, 1 additional patient achieved a clinically significant improvement in FIM at discharge (NNT 13, 95% CI 7 to 71) (Table 3).

**Table 2:** Primary outcomes

**Outcome**	**Groups**								**Difference between groups**		
	**Admission**		**Discharge**		**Month 6**		**Month 12**		**Discharge, Int - Con**	**Month 6, Int - Con**	**Month 12, Int - Con**
	**Int**	**Con**	**Int**	**Con**	**Int**	**Con**	**Int**	**Con**			
FIM total	84 (19)	84 (20)	106 (18)	104 (20)	109 (17)	107 (19)	109 (17)	108 (19)	2.3 (0.5 to 4.1)*; *P* = 0.01	2.0 (0.0 to 4.0)*; *P* = 0.05	1.3 (−0.9 to 3.5); *P* = 0.24
Mobility score	16 (7)	16 (7)	26 (6)	25 (7)	28 (7)	28 (7)	29 (7)	28 (8)	1.0 (0.3 to 1.6)*; *P* = 0.006	0.6 (−0.2 to 1.3); *P* = 0.14	0.6 (−0.3 to 1.4); *P* = 0.19
Self-care score	27 (8)	27 (8)	36 (7)	35 (8)	37 (7)	36 (8)	37 (8)	37 (8)	0.6 (−0.2 to 1.3); *P* = 0.13	0.8 (−0.1 to 1.7); *P* = 0.09	0.4 (−0.6 to 1.4); *P* = 0.41
EQ-5D VAS (0 to 100)	57 (21)	56 (22)	71 (19)	70 (17)	71 (20)	70 (21)	71 (20)	70 (19)	1.1 (−1.0 to 3.2); *P* = 0.30	0.3 (−2.2 to 2.8); *P* = 0.84	0.8 (0 to 1.6); *P* = 0.59
EQ-5D utility index	0.32 (0.35)	0.37 (0.35)	0.65 (0.28)	0.62 (0.28)	0.63 (0.36)	0.61 (0.37)	0.64 (0.39)	0.64 (0.34)	0.04 (0.01 to 0.07)*; *P* = 0.009	0.03 (−0.01 to 0.08); *P* = 0.15	0.01 (−0.04 to 0.05); *P* = 0.77

Intervention (Int) n = 496, control (Con) n = 500; intervention = Monday to Saturday rehabilitation, control = Monday to Friday rehabilitation). Mean (SD) of groups and mean (95% CI) difference between groups at admission, discharge, 6 months and 12 months are shown.

*P ≤0.05.

*EQ-5D* EuroQoL five dimensions questionnaire, *FIM* functional independence measure, *VAS* visual analog scale.

**Table 3:** Numbers of participants (absolute risk %) who had achieved a minimally clinically important difference in functional independence and health-related quality of life from admission to assessment at discharge, 6 months and 12 months

**Outcome**	**Time point**	**Intervention**	**Control**	**Relative risk difference (95% CI)**	**Number needed to treat (95% CI)**
Functional independence (FIM)	Discharge	256 (52)	220 (44)	1.17 (1.03 to 1.34)*	13 (7 to 71)
	6 months	274 (55)	261 (52)	1.06 (0.94 to 1.19)	33 (−32 to 11)
	12 months	284 (57)	266 (53)	1.07 (0.96 to 1.20)	25 (−47 to 10)
Health-related quality of life (EQ-5D)	Discharge	262 (53)	222 (44)	1.18 (1.04 to 1.34)*	12 (7 to 45)
	6 months	287 (58)	243 (49)	1.19 (1.06 to 1.34)*	11 (7 to 33)
	12 months	289 (58)	262 (52)	1.11 (1.00 to 1.24)	17 (−326 to 8)

Intervention = Monday to Saturday rehabilitation, control = Monday to Friday rehabilitation.

*Statistically significant.

*EQ-5D* EuroQoL five dimensions questionnaire.

#### Health-related quality of life

Participants in the intervention group had higher EQ-5D utility index scores (MD 0.04, 95% CI 0.01 to 0.07, *P* = 0.009) on discharge and possibly at 6 months (MD 0.03, 95% CI −0.01 to 0.08, *P* = 0.15) but not at 12 months (MD 0.01, 95% CI −0.04 to 0.05, *P* = 0.77) when compared to the control group. Participants in both groups scored similarly on the EQ-5D VAS at discharge, 6 months and 12 months (Table 2). Participants who received Monday to Saturday rehabilitation were 18% more likely to achieve a clinically significant improvement in health-related quality of life utility index score at discharge (RR = 1.18, 95% CI 1.04 to 1.34) than participants who received Monday to Friday rehabilitation. This difference was maintained at 6 months and possibly at 12 months. For every 12 patients provided with the intervention, 1 additional patient achieved a clinically significant improvement in EQ-5D at discharge (NNT 12, 95% CI 7 to 45) (Table 3).

#### Length of stay

The intervention group may have had a shorter length of stay by 2 days (95% CI 0 to 4, *P* = 0.1) compared to the control group, with length of stay reduced from a mean of 23 (SD 20) days to 21 (SD 16) days. Few participants were discharged on a weekend day; 15 participants in the intervention group and 11 participants in the control group.

#### Secondary outcomes

There were no significant differences between the groups in PC-PART, modified Motor Assessment Scale, or timed up and go test at discharge (Table 4). The intervention group may have had a faster walking speed on discharge compared to the control group (MD 0.03, 95% CI 0.00 to 0.06, *P* = 0.09). In total, 88% of participants who were living independently in the community prior to their admission returned to their previous living accommodation; there were no differences between groups in terms of discharge destination (RR = 0.98, 95% CI 0.93 to 1.03) or need for follow-up outpatient or community allied health services (RR = 0.96, 95% CI 0.90 to 1.01).

**Table 4:** Secondary outcomes

**Outcome (number of participants in analysis)**	**Groups**				**Difference between groups**	
	**Admission**		**Discharge**		**Intervention - control**	***P*value**
	**Intervention**	**Control**	**Intervention**	**Control**		
PC-PART (0 to 43) (n = 963)	13(8)	14(8)	2(4)	3(6)	−0.3 (−0.9 to 0.3)	0.30
10-m walk test (m/s) (n = 694)	0.52(0.31)	0.48(0.28)	0.73(0.30)	0.68(0.29)	0.03 (0.00 to 0.06)	0.09
Timed up and go test (s) (n = 677)	42(36)	39(24)	24(21)	24(13)	−1 (−3 to 1)	0.32
MMAS (0 to 48) (n = 151)	25(15)	27(14)	34(14)	34(12)	1.9 (−0.4 to 4.2)	0.10

Intervention = Monday to Saturday rehabilitation, control = Monday to Friday rehabilitation. Mean (SD) of groups and mean (95% CI) difference between groups on discharge are shown.

*MMAS* Modified Motor Assessment Scale for participants with stroke (a lower score indicates a higher level of impairment), *PC-PART* Personal Care Participation Assessment and Resource Tool (a lower score indicates more independence with personal care tasks).

### Adverse events

No serious adverse events occurred during the additional Saturday rehabilitation. There were a total of 240 adverse events reported during inpatient rehabilitation. Adverse events included non-injurious falls (intervention group n = 50, control group n = 70) and minor medical issues such as skin tears (intervention group n = 42, control group n = 41). No adverse events were classified as causing serious harm and two were classified as causing moderate harm (intervention group n = 1, control group n = 1). Participants in the intervention group had an observed adverse event rate of 19% less than participants in the control group (incidence rate ratio = 0.81, 95% CI 0.61 to 1.08), but this did not reach statistical significance.

## Discussion

During inpatient rehabilitation, providing additional allied health services helped patients to get better quicker. Patients who received additional Saturday rehabilitation were discharged at a higher level of functional independence and with higher health-related quality of life than those who received Monday to Friday rehabilitation despite being discharged home sooner. The likely reduction in length of stay did not come at the expense of poorer discharge outcomes. Participants who received Monday to Saturday rehabilitation were just as likely to be discharged home (and not to a residential facility) and just as likely to need follow-up outpatient services on discharge compared to those in the control group. These results confirm findings from a systematic review about the benefits of providing additional therapy [37] and add to previous research on the provision of additional weekend rehabilitation services [13,14] by providing evidence from an adequately-powered, prospective, randomized controlled trial including 12-month follow-up.

In this trial, patients who received Monday to Saturday rehabilitation did not receive a great deal more rehabilitation (mean 53 minutes, 13% extra) than patients who received Monday to Friday rehabilitation but this additional rehabilitation did improve outcomes. The amount of additional rehabilitation was somewhat less than the expected, which could be due to missed sessions of therapy as a consequence of feeling unwell, day leave on a Saturday or because patients were admitted late in the week and had not been recruited, assessed and randomized to be scheduled for weekend therapy. However, the additional rehabilitation provided did improve outcomes.

Rehabilitation in the form of physiotherapy and occupational therapy typically focused on task specific training and discharge planning. This additional rehabilitation alone may have been enough to improve outcomes if patients made gains during the extra sessions of therapy. However, other factors may have also contributed to improved outcomes. Patients who received Saturday rehabilitation did not have a 2-day break in therapy, which may have reduced time for functional decline due to inactivity. Analysis of the physical activity levels of a subset of participants in the current trial found that those receiving Saturday rehabilitation were more physically active on both days of the weekend compared to those who received Monday to Friday rehabilitation [29]. In addition, higher levels of physical activity during rehabilitation were associated with higher levels of functional independence on discharge and shorter length of stay [30]. Therefore, the additional physical activity associated with weekend rehabilitation may have contributed to improving outcomes. In a qualitative study on another subset of participants in the current trial, additional Saturday rehabilitation was reported to change patient perceptions of what weekends in rehabilitation were for [31]. Patients who received Saturday rehabilitation expected to be working towards their rehabilitation goals over the weekend while those who received Monday to Friday rehabilitation expected to rest over the weekend. These changed patient expectations may have contributed to improved outcomes with Monday to Saturday rehabilitation in the current trial.

We also found that benefits in functional independence and health-related quality of life gained from additional weekend rehabilitation may have been maintained for up to 6 months post discharge suggesting that the more successful outcome achieved during rehabilitation may have had ongoing effects. Most improvement occurred during inpatient rehabilitation when therapy was being provided with only relatively small gains following discharge (Table 2). Previous trials on functional outcomes following rehabilitation for stroke [38-40] and hip fracture [41] also found that most functional gains were made between admission and discharge from rehabilitation with results maintained (but not improved upon) at 6-month or 12-month follow-up. Therefore, it cannot be assumed that patients are going to get better on their own at home following discharge from rehabilitation, reinforcing the importance of maximizing functional gains during the inpatient rehabilitation period.

There were no significant differences between groups in terms of most secondary outcomes, including the timed up and go test, PC-PART and the modified Motor Assessment Scale. This may reflect the goals of rehabilitation where interventions were focused on improving overall functional independence for discharge back to living in the community rather than specific activities such as balance, walking speed or upper limb function.

Recent debate has highlighted the issue of weekend healthcare provision and the benefits and difficulties in providing weekend healthcare [8,9]. Our trial demonstrated that providing weekend rehabilitation services, at least on a Saturday, improved functional independence and health-related quality of life and reduced length of stay, which may have clinical implications for both patients and health services. These results may also be applicable to settings and cultures where rehabilitation is currently provided 5 days a week even if Saturday may be a usual work day as the Saturday rehabilitation in this trial reflects an additional day, or a sixth day of rehabilitation. Patients may not have to wait for as long for a rehabilitation bed, and can return home sooner with better function to resume their usual activities in the community. However, one of the key concerns about providing weekend care is the question of who will pay for the additional services [8,42]. Because intervention group participants achieved better clinical outcomes at discharge despite likely having a shorter length of stay in our trial, health service providers may be able to treat more patients throughout the year which may lead to cost advantages. A formal economic evaluation is being conducted separately alongside the current trial.

This trial included participants with a variety of health conditions requiring rehabilitation, non-English speaking participants, and participants with cognitive impairment making the results generalizable to many metropolitan inpatient rehabilitation facilities. A limitation is that subgroup analyses were not planned or completed, therefore we do not know if the results are particularly applicable to patients with certain diagnoses. However, our trial was not powered for subgroup analyses and such *post hoc* analyses are discouraged [43]. In addition, we took a health service perspective about staffing a service rather than providing therapy based on a specific diagnosis. Risk of bias was minimized through concealed, random allocation of participants and the use of blinded assessors throughout the clinical trial and follow-up period; however, patients, therapists and other clinical staff were not blinded to group allocation. Follow-up measurements at 6 and 12 months were completed by telephone and not face-to-face which may have introduced error; however, all project officers were credentialed to administer the FIM, there were high compliance rates, and there is evidence that telephone administration of the FIM and EQ-5D is suitable for older adults following hospitalization [44,45]. Another potential limitation is that the additional rehabilitation was only provided by physiotherapists and occupational therapists. We acknowledge the important contributions of other members of the rehabilitation team such as social workers, podiatrists and dietitians. However, we chose physiotherapy and occupational therapy as they are the most commonly required and provided interventions during rehabilitation [2].

## Conclusions

Providing additional allied health services (physiotherapy and occupational therapy) on Saturdays during inpatient rehabilitation helped patients to regain their functional independence faster. Future research could focus on the dose–response relationship of additional weekend rehabilitation services, and explore whether the additional amount of rehabilitation therapy or reducing the consecutive amount of time without rehabilitation therapy improved outcomes.

## Abbreviations

ANCOVA: Analysis of covariance; CI: confidence interval; EQ-5D: EuroQoL five dimensions questionnaire; EQ-VAS: EuroQoL questionnaire visual analog scale; FIM: Functional independence measure; MD: Mean difference; PC-PART: Personal Care Participation Assessment and Resource Tool; RR: Risk ratio.

## Competing interests

The authors declare that they have no competing interests.

## Authors’ contributions

NS, NB, JW and NT were responsible for study concept and study design and CP contributed to study design. CP and NB were responsible for data collection. CP, NS and NT were responsible for data analysis and data interpretation. CP wrote the first draft of the manuscript and CP, NS, NB, JW and NT contributed to writing the final manuscript. CP and NT are the guarantors. All authors read and approved the final manuscript.
