# Correction: Additional Saturday rehabilitation improves functional independence and quality of life and reduces length of stay: a randomized controlled trial

**DOI:** 10.1186/1741-7015-11-262

**Published:** 2013-12-20

**Authors:** Casey L Peiris, Nora Shields, Natasha K Brusco, Jennifer J Watts, Nicholas F Taylor

**Affiliations:** 1 Department of Physiotherapy, La Trobe University, Melbourne, Victoria, Australia; 2 Allied Health Clinical Research Office, Eastern Health, Box Hill, Victoria 3128, Australia; 3 Allied Health Learning and Research Unit, Northern Health, Bundoora, Victoria, Australia; 4 Physiotherapy Services, Cabrini Health, Malvern, Victoria, Australia; 5 School of Health and Social Development, Faculty of Health, Deakin University, Burwood, Victoria, Australia

## Authors’ correction note

On reviewing our recently published trial in BMC Medicine [1], we realised that there were some minor errors in the demographic data reported in Table 1 and in 2 sentences of the accompanying text. Specifically, our sample comprised 365 men, not 359 as reported, and there were some very minor differences in the numbers of participants reported in each diagnostic category. The main contributing factors for the minor errors were misinterpretation of gender neutral first names, and grouping of the diagnostic codes assigned during data collection for reporting. We believe these changes do not affect the results or conclusions of our study, and are confident in the processes we employed (full double data entry by two independent teams) to ensure the integrity of the rest of our data. Table 1 has been corrected and the first two sentences in the accompanying text in the methods when describing the participants should read: Participants had a mean (SD) age of 74 (13) years and 631 (63%) were women (Table 1). A total of 581 (58%) participants were admitted with an orthopedic diagnosis, 203 (20%) with a neurological diagnosis and 212 (21%) participants were admitted with other disabling impairments. A total of 94% of participants were living independently in the community prior to their acute hospital admission.

**Table 1:** Baseline characteristics

**Characteristic**	**Randomized (n = 996)**	
	**Intervention**	**Control**
	**(n = 496)**	**(n = 500)**
Age in years, mean (SD)	75 (13)	74 (13)
Age group, n (%)		
≤ 59 years	63 (13)	72 (14)
60 to 79 years	236 (48)	234 (47)
≥ 80 years	197 (40)	194 (39)
Gender, n male (%)	189 (38)	176 (35)
Diagnosis category, n (%)		
Stroke	81 (16)	79 (16)
Other neurological conditions	19 (4)	24 (5)
Orthopedic conditions	284 (57)	297 (59)
Pain syndromes	24 (5)	19 (4)
Cardiac/Pulmonary	25 (5)	23 (5)
Other disabling impairments	63 (13)	58 (12)
Functional independence (FIM)		
Total, mean (SD)	83 (20)	83 (21)
Mobility component, mean (SD)	16 (7)	16 (7)
Self-care component, mean (SD)	27 (8)	27 (8)
Cognitive component, mean (SD)	31 (6)	31 (6)
Health-related quality of life		
EQ-5D utility index, mean (SD)	0.32(0.35)	0.37(0.35)
Visual analog scale (0 to 100 mm), mean (SD)	57(21)	56(22)
Charlson comorbidity index, mean (SD)	1 (1)	1 (1)
Living independently in the community prior to admission, n (%)	466 (94)	466 (93)

Intervention = Monday to Saturday rehabilitation, control = Monday to Friday rehabilitation.

*EQ-5D* EuroQoL five dimensions questionnaire.

This is a Correction article on http://www.biomedcentral.com/1741-7015/11/198.

## Corrected text (page 4: Participants, first 2 sentences)

Please replace: Participants had a mean (SD) age of 74 (13) years and 637 (64%) were women (Table 1). A total of 579 (58%) participants were admitted with an orthopedic diagnosis, 203 (20%) with a neurological diagnosis and 214 (21%) participants were admitted with other disabling impairments.

With the amended text:

Participants had a mean (SD) age of 74 (13) years and 631 (63%) were women (Table 1). A total of 581 (58%) participants were admitted with an orthopedic diagnosis, 203 (20%) with a neurological diagnosis and 212 (21%) participants were admitted with other disabling impairments. A total of 94% of participants were living independently in the community prior to their acute hospital admission.
